# A Single Dose of Goji Berries Does Not Affect Postprandial Energy Expenditure and Substrate Oxidation in Healthy, Overweight Men

**DOI:** 10.1155/2019/4057143

**Published:** 2019-02-24

**Authors:** José J. van den Driessche, Jogchum Plat, Guy Plasqui, Ronald P. Mensink

**Affiliations:** Department of Nutrition and Movement Sciences, NUTRIM School for Nutrition and Translational Research in Metabolism, Maastricht University Medical Center+ (MUMC+), Maastricht, Netherlands

## Abstract

**Background and Aim:**

Increasing energy expenditure is an effective strategy for the prevention of obesity. In this respect, *Lycium barbarum* (goji berry) is of interest, as it has been shown to increase postprandial oxygen consumption. Although this suggests that energy expenditure was also increased, energy expenditure and substrate oxidation can only be assessed accurately when both oxygen consumption and carbon dioxide production are measured. We therefore investigated the effects of a single dose of *Lycium barbarum* fruit on postprandial energy expenditure and substrate oxidation in a randomized, double-blind crossover trial. In addition, markers of lipid and glucose metabolism were measured.

**Methods:**

Seventeen healthy, overweight men received in a random order a meal containing 25 grams of dried *Lycium barbarum* fruit or a control meal matched for caloric content and macronutrient composition. Energy expenditure and the respiratory quotient were determined using indirect calorimetry before and up to 4 hours after meal intake. Blood was sampled before and after meal intake at regular intervals for analyses of plasma glucose, serum triacylglycerol, and free fatty acid concentrations.

**Results:**

Energy expenditure significantly increased after the *Lycium barbarum* and control meal, but no differences were found between the meals (*p*=0.217). Postprandial changes in respiratory quotient (*p*=0.719) and concentrations of glucose (*p*=0.663), triacylglycerol (*p*=0.391), and free fatty acids (*p*=0.287) were also not affected by *Lycium barbarum* intake.

**Conclusions:**

A single dose of *Lycium barbarum* does not affect postprandial energy expenditure, substrate oxidation, and markers for lipid and glucose metabolism in healthy, overweight men.

## 1. Introduction

One of the main risk factors for the onset of cardiovascular diseases (CVDs) and type II diabetes mellitus is obesity, which develops when energy intake exceeds energy expenditure [1]. Thus, increasing energy expenditure is a promising strategy to prevent obesity, thereby lowering the risk to develop CVDs and type II diabetes mellitus. In addition, impaired fasting and postprandial fat oxidation have been linked to an increased risk for weight gain and obesity [2, 3]. In this light, foods affecting energy expenditure and fat oxidation are of interest.

Berries from the plant *Lycium barbarum* (goji berry, wolfberry) originate from Asia and have recently gained popularity in European countries [4]. Major constituents of goji berries include polysaccharides, specifically referred to as *Lycium barbarum* polysaccharides (LBP), carotenoids (mainly zeaxanthin), and vitamins [5]. *Lycium barbarum* has been consumed in Asia as part of traditional medicine for decades due to its potential beneficial effects on, among others, the development of CVDs and type II diabetes mellitus. It has also been suggested that the goji berry stimulates metabolism. Indeed, animal studies have indicated that expression of genes related to energy metabolism, such as UCP-1 and PGC1*α*, was elevated after *Lycium barbarum* intake [6]. Unfortunately, energy expenditure was not measured in these experiments. However, the effect of *Lycium barbarum* on energy metabolism has been investigated in one human trial. In that study, it was found that a single dose of *Lycium barbarum* increased postprandial oxygen consumption in healthy men and women [7]. Although this suggests that energy expenditure was increased, energy expenditure and substrate oxidation can only be assessed accurately when both oxygen consumption and carbon dioxide production are measured. The main objective of the current study was therefore to investigate the effects of a single dose of 25 grams dried *Lycium barbarum* fruit as part of a meal on postprandial energy expenditure and substrate oxidation in healthy, overweight men. In addition, effects on markers for postprandial lipid and glucose metabolism were studied, since postprandial hyperglycemia and hypertriglyceridemia are established risk factors for CVD [8, 9]. Overweight subjects were studied, as they are at increased risk to develop obesity and subsequently CVDs and type II diabetes mellitus and might therefore benefit most from interventions targeting energy expenditure.

## 2. Subjects and Methods

### 2.1. Study Population

Apparently healthy overweight men were recruited using advertisement in local newspapers, online advertisements, and posters in university buildings and the hospital. Furthermore, subjects that had already participated in earlier studies from our department were approached. Men were invited for a screening visit if they met the following criteria: aged between 18 and 65 years, BMI between 25 and 30 kg/m^2^, nonsmoking, no use of anticoagulants or medications known to affect lipid or glucose metabolism, no conditions that might interfere with study outcomes, stable body weight (≤3 kg weight loss or gain in the past 3 months), no participation in another biomedical study during the past month, and no abuse of drugs or alcohol. During a screening visit, fasting blood samples were taken for analysis of serum lipids and plasma glucose. In addition, height and weight were measured. Eighteen subjects were enrolled. These subjects had fasting serum triacylglycerol concentrations below 2.2 mmol/L and no elevated fasting serum cholesterol (<8.0 mmol/L) or plasma glucose (<7.0 mmol/L) concentrations. All subjects signed informed consent before the screening visit. This study was approved by the medical ethical committee of Maastricht University Medical Centre+ (MUMC+) and registered at clinicaltrials.gov as NCT02779985.

### 2.2. Study Design

A randomized, double-blind, crossover study with two treatments was carried out. For this, subjects visited the university during two occasions separated by a washout period of at least 7 days. On the day preceding each test day, subjects were asked to abstain from alcohol consumption, exercise, and caffeine consumption (from 12.00 PM onwards) and to consume a standardized meal in the evening. Subjects were instructed to select a ready-made meal with a fixed macronutrient composition (30–40% fat, 40–50% carbohydrates, and 13–16% proteins) from a list and to consume the same meal the evening before both test days to eliminate potential effects of the previous meal [10].

After an overnight fast (from 08.00 PM), subjects came to the university by public transport or by car to limit physical activities in the morning as much as possible. Before the start of the measurements, subjects rested for 15 minutes in supine position, and an intravenous catheter was placed for blood withdrawal. Next, indirect calorimetry was performed for 30 minutes and a blood sample was drawn (T0). Subjects were then asked to consume one of the two test meals in three equal parts divided over 10 minutes. After meal intake, indirect calorimetry measurements were continued for another 140 minutes. Next, the hood was removed, and after a 20-minute break, subjects were allowed to stroll around; measurements were continued for another 40 minutes (T160–T200). The last hour of the test days consisted of another 20-minute break and 40 minutes of indirect calorimetry measurements (T220–T260). Blood samples were taken 15 min (T15), 30 min (T30), 45 min (T45), 60 min (T60), 90 min (T90), 120 min (T120), 180 min (T180), and 240 min (T240) after meal intake. A validated food frequency questionnaire was used to assess habitual food intake over the past month. Calorie content and macronutrient composition (Supplemental Table 1) was calculated using the Dutch food composition table (NEVO). Throughout the study, subjects were asked not to change their habitual diet and exercise pattern and to record in a study diary their alcohol consumption and any changes in health status.

### 2.3. Test Meals

At the two test days, subjects received a meal containing 25 grams of dried *Lycium barbarum* fruit (82 kcal, 0.9 g fat, 13.3 g carbohydrates, and 3.3 g protein per 25 grams; Superfood.nl, the Netherlands) or a control meal matched for macronutrient composition and energy content (Table 1). The amounts of carbohydrates, fat, and proteins provided by *Lycium barbarum* fruit were in the control meal derived from plant-based sources. The *Lycium barbarum* and control meals had similar energy contents (684 kcal and 683 kcal, respectively) and macronutrient composition (55 En% fat, 32 En% carbohydrate, 12 En% protein vs. 55 En% fat, 33 En% carbohydrate, and 12 En% protein). Meals contained over 40 grams of fat to trigger a postprandial triacylglycerol response [11]. The test meals, which were prepared by a research dietician, were flavored with caramel and presented in red, masked cups to blind the subject and the investigator.

### 2.4. Indirect Calorimetry

Oxygen consumption (VO_2_) and carbon dioxide production (VCO_2_) were measured during fasting and postprandial conditions using a ventilated hood system (Omnical, Maastricht University, Maastricht, the Netherlands). Calibration of the indirect calorimeter was performed automatically every 30 min with span gas (18% O_2_ and 0.8% CO_2_) and nitrogen gas (100%). Validation of the system was performed regularly during the study with a methanol combustion test. VO_2_, VCO_2_, and respiratory quotient (RQ) were averaged over 20 minutes at baseline (T0) and 10–30 min (T20), 30–50 min (T40), 50–70 min (T60), 70–90 min (T80), 90–110 min (T100), 110–130 min (T120), 170–190 min (T180), and 230–250 min (T240) after meal intake. Energy expenditure was calculated from VO_2_ and VCO_2_ data using the formula of Weir [12]. Fat and carbohydrate oxidation were calculated using stoichiometric equations [13].

### 2.5. Biochemical Analysis

NaF-containing vacutainer tubes (Becton, Dickinson and Company, Franklin Lakes, NJ, USA) were placed on ice immediately after blood withdrawal. Tubes were centrifuged within 30 minutes at 1300 × g for 15 minutes at 4°C. Serum separator tubes (Becton, Dickinson and Company) were allowed to clot for 30–60 minutes at room temperature after withdrawal and centrifuged at 21°C for 15 minutes at 1300 × g. All plasma and serum samples were directly frozen in liquid nitrogen and stored at −80°C until analysis.

At all time points, NaF plasma was used for analysis of glucose (Horiba ABX, Montpellier, France) and serum for analysis of free fatty acids (WAKO Chemicals GmbH, Neuss, Germany). Serum triacylglycerol concentrations corrected for free glycerol (Sigma-Aldrich Corp., St. Louis, MO, USA) were measured at T0, T30, T60, T120, T180, and T240.

### 2.6. Statistical Analysis

It was calculated that a sample size of 18 subjects was needed to detect a difference of 0.18 kJ/min with a power of 80% and a within-subject variation of 0.25 kJ/min [14].

All data are presented as mean ± SD. Differences between test days in fasting values were compared using a paired-sample *T*-test. Postprandial changes from baseline were analyzed using linear mixed models with meal and time as fixed factors and meal ∗ time as an interaction term. The interaction term was not significant in any of the models and therefore removed from all models. If the factor time was significant, time points were compared to baseline using post hoc tests with Bonferroni correction. The incremental area under the curve (iAUC), defined as the area above baseline values, was calculated with the trapezoidal rule [15] for the 4 hours after meal intake. The decremental area under the curve (dAUC), defined as the area below baseline values, was calculated likewise. iAUCs and dAUCs were not normally distributed, as was apparent from the Shapiro–Wilk test. Therefore, values are presented as medians and ranges, and differences between test meals were compared using nonparametric tests. *p* values < 0.05 were considered statistically significant. Statistical analyses were performed with SPSS 21.0 for Mac (IBM Corp., Armonk, NY, USA).

## 3. Results

### 3.1. Subject Characteristics

Eighteen subjects were included and completed the study (Supplemental Figure 1). One of the subjects was excluded from analysis due to clear absence of a postprandial response in all parameters measured during one of the test days. Average age of the remaining 17 subjects was 59.5 ± 5.4 years. Subjects were overweight with an average BMI of 27.2 ± 1.4 kg/m^2^. Inspection of the diaries did not reveal any protocol deviations that may have affected the results. Baseline characteristics of the 17 subjects who completed the study are presented in Table 2.

### 3.2. Energy Expenditure

**Figure 1:**
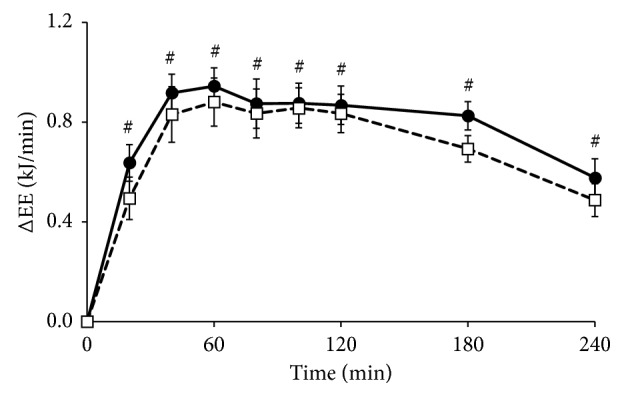
Mean changes (±SEM) in energy expenditure (EE) following the *Lycium barbarum* meal (☐) and the control meal (•) in 17 healthy overweight men. Data were analyzed using linear mixed models. After Bonferroni correction, the factor time was significantly different from baseline values at all time points (*p* < 0.001) (#).

Baseline energy expenditure did not differ between the two visits (*p*=0.709, data not shown). After meal intake, energy expenditure increased significantly (*p* < 0.01 for factor time; Figure 1). No difference was found between the *Lycium barbarum* and control meals (*p*=0.217 for factor meal). The iAUC over 4 hours was also not significantly different between the two meals (*p*=0.113, Supplemental Table 2).

VO_2_ and VCO_2_ (Supplemental Figure 2) did not differ between the *Lycium barbarum* and control meals at baseline (*p*=0.673 and *p*=0.885, respectively) and after meal intakes (*p*=0.212 and *p*=0.431, respectively).

### 3.3. Respiratory Quotient and Substrate Oxidation

**Figure 2:**
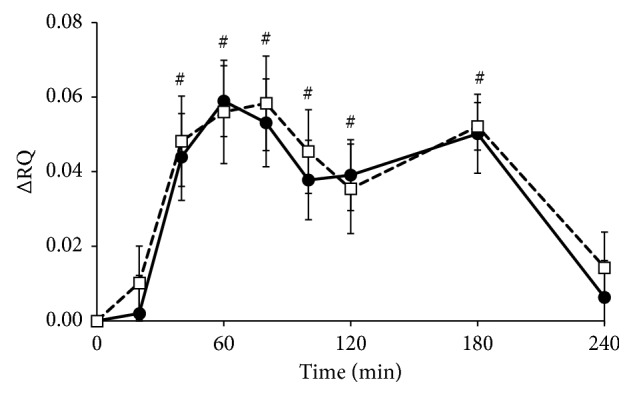
Mean changes (±SEM) in respiratory quotient (RQ) following the *Lycium barbarum* meal (☐) and the control meal (•) in 17 healthy overweight men. Data were analyzed with linear mixed models. Significant effects compared to baseline (*p* < 0.001 with Bonferroni correction) (#) were found for factor time.

The RQ at baseline did not differ between the test days (*p*=0.845, data not shown) but increased 40 minutes after meal intake and returned to baseline after 240 minutes (*p* < 0.01 for factor time; Figure 2). Changes over time were not affected by the intake of *Lycium barbarum* (*p*=0.719 for factor meal and *p*=0.523 for iAUC, Supplemental Table 2).

Fat and carbohydrate oxidation (Supplemental Figure 3) did not differ between the *Lycium barbarum* and control meals at baseline (*p*=0.865 and *p*=0.991, respectively) and after meal intake (*p*=0.549 and *p*=0.909 for factor meal, respectively).

### 3.4. Glucose and Lipid Metabolism

**Figure 3:**
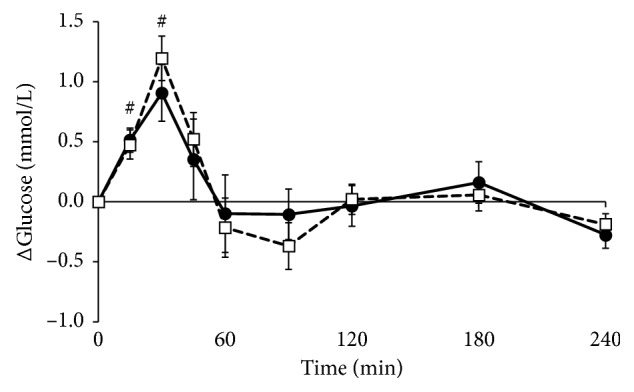
Mean changes (±SEM) in plasma glucose concentrations following the *Lycium barbarum* meal (☐) and the control meal (•) in 17 healthy overweight men. Data were analyzed using linear mixed models. Significant effects were found for factor time (*p* < 0.001 with Bonferroni correction) (#) compared to baseline.

Plasma glucose concentrations at baseline did not differ between the two visits (*p*=0.724, data not shown). Glucose concentrations increased significantly after meal intake and returned to baseline after 45 minutes (*p* < 0.01 for factor time; Figure 3) but did not differ between meals (*p*=0.663 for factor meal and *p*=0.332 for iAUC, Supplemental Table 2).

**Figure 4:**
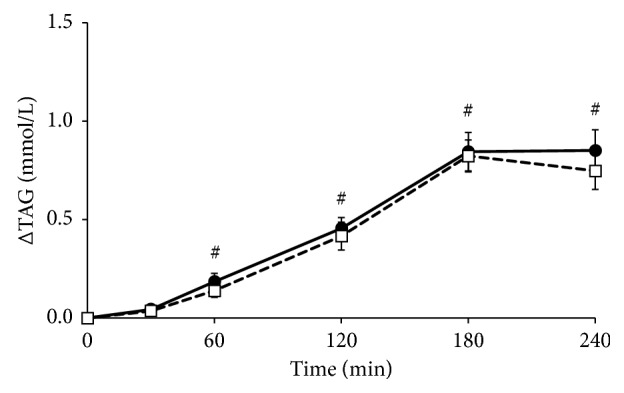
Mean changes (±SEM) in serum triacylglycerol (TAG) concentrations following the *Lycium barbarum* meal (☐) and the control meal (•) in 17 healthy overweight men. Data were analyzed using linear mixed models. Significant effects (*p* < 0.05, with Bonferroni correction) were found for factor time (#) compared to baseline.

**Figure 5:**
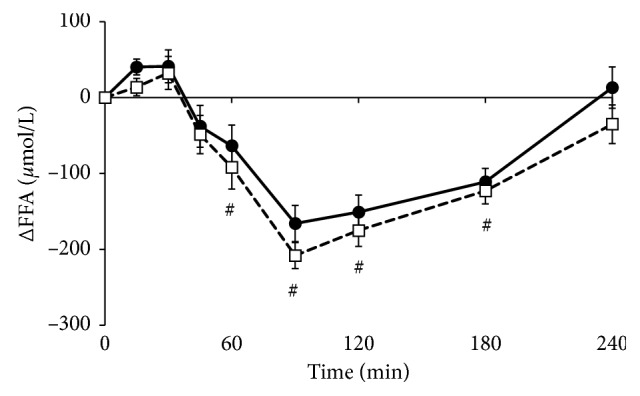
Mean changes (±SEM) in serum-free fatty acids (FFA) concentrations following the *Lycium barbarum* meal (☐) and the control meal (•) in 17 healthy overweight men. Data were analyzed using linear mixed models. Significant effects (*p* < 0.001, with Bonferroni correction) were found for factor time (#) compared to baseline.

Serum triacylglycerol and FFA concentrations at baseline did not differ between test days (*p*=0.914 and *p*=0.330, respectively). Sixty minutes after meal intake, triacylglycerol concentrations were increased and remained elevated throughout the test days (*p* < 0.01 for factor time; Figure 4). However, effects between the meals (*p*=0.391 for factor meal and *p*=0.287 for iAUC, Supplemental Table 2) were not significantly different. FFA concentrations significantly decreased within 60 minutes after meal intake and returned to baseline concentrations after 240 minutes (*p* < 0.01 for factor time; Figure 5). No differences were found between the meals (*p*=0.133 for factor meal and *p*=0.723 for dAUC, Supplemental Table 2).

## 4. Discussion

In this study, we found no effects of a single dose of 25 g dried *Lycium barbarum* fruit on postprandial energy expenditure and substrate oxidation in healthy, overweight men. This contrasts findings in animal studies indicating that *Lycium barbarum* intake affected energy metabolism, as suggested by increased expression of genes involved in energy metabolism and increased brown adipose tissue activity [6]. In two studies [6, 16], the body weight of mice and rats fed with a high-fat diet also decreased after intake of *Lycium barbarum* extracts, whereas food intake was not altered. In these animal trials, water-soluble polysaccharides (LBP) were examined, which are thought to be the main bioactive component of goji berries [4]. However, energy expenditure was not measured in these experiments. In one human study with 8 healthy overweight men and women [7], effects of single doses of *Lycium barbarum* on postprandial oxygen consumption were examined. Compared to placebo, only the highest dose of 120 ml goji berry juice significantly increased oxygen consumption 1 and 4 hours after meal intake. One of the limitations of that trial, as also discussed by the authors, was the small sample size. Additionally, only oxygen consumption was measured. To assess energy expenditure and substrate oxidation accurately, both oxygen consumption and carbon dioxide production need to be measured, as we did. Nonetheless, as already mentioned, no effects were found, also not on oxygen consumption. We have no obvious explanation for these discrepancies in results. Differences in subject characteristics may have played a role. Although in both trials healthy overweight subjects participated, participants in the trial of Amagase and Nance were younger (34.5 ± 7 years), while both men and women were included.

The single dose of 25 grams of dried *Lycium barbarum* fruit used in the current study fits within the range of 15–30 grams of the dried fruit frequently used in traditional Asian medicine [5]. In the study of Amagase and Nance, 120 ml of LBP-standardized goji berry juice was used, corresponding to the amount of LBP found in 150 grams of fresh berries. This dose is comparable to the amount of dried fruit used in our trial. Although the *Lycium barbarum* provided in both trials was processed differently, it is unlikely that this explains the difference in results. Different preparations of *Lycium barbarum*, such as powders or juices [17, 18] and extracts isolated from the dried fruit, have been found to be biologically active [19], suggesting that potentially active constituents are not lost during drying.

In both trials, *Lycium barbarum* was provided with a meal, but caloric intake as well as macronutrient composition of the meals was different between the two studies. In the study of Amagase and Nance, the caloric content of the highest dose of goji berry juice combined with the meal was 260 kcal less than our meal. The meal, excluding the goji berry juice, provided 55% of the energy from carbohydrates, whereas our meal provided 55% of the energy from fat. However, whether differences in meal composition can explain the discrepancies in results warrants further study.

No long-term studies have been performed investigating the effects of *Lycium barbarum* intake on measures of energy expenditure. However, some studies have measured changes in body weight, which could serve as a proxy for energy balance. In mice, body weight was reduced after long-term *Lycium barbarum* intake [6, 20]. In humans, however, a recent meta-analysis did not find any effects of *Lycium barbarum* intake on body weight [21]. In the five trials included, a total of 366 subjects were supplemented with *Lycium barbarum* or placebo for 14 days to 3 months [17, 18, 22–24]. In none of these trials, food intake was controlled. Though suggestive, from this meta-analysis, it cannot definitely be concluded that energy expenditure did not change, since other factors might have influenced body weight as well in these trials.

Another question is whether *Lycium barbarum* may reduce cardiovascular disease risk by improving postprandial lipid and glucose metabolism. Only one other study has addressed this question. This was, however, an intervention trial with intake of *Lycium barbarum* over three months instead of a single dose. Nevertheless, postprandial glucose concentrations were lowered in this trial [24]. In rats, LBP administration for four weeks stimulated translocation and activation of glucose transporter isoform 4 (GLUT4) in adipocytes and lowered glucose concentrations [25]. Furthermore, LBPs have been shown to reduce intestinal glucose uptake in Caco-2  cells [26]. In our study, no changes in postprandial glucose concentrations were found. One may argue that a single dose of *Lycium barbarum* might not be sufficient to induce changes in glucose concentrations, whereas repeated intake does. In the study of Cai et al. [24], no effects of repeated *Lycium barbarum* consumption were found on postprandial triacylglycerol concentrations, which is in line with the results from our single dose study.

In summary, our study indicates that a single dose of *Lycium barbarum* does not influence postprandial energy expenditure and substrate oxidation in healthy, overweight men. Furthermore, *Lycium barbarum* intake did not affect postprandial plasma glucose, serum-free fatty acids, and triacylglycerol concentrations.

## Figures and Tables

**Table 1:** Macronutrient composition of the mixed test meals.

	*Lycium barbarum* meal^*∗*^	Placebo meal
Energy (kcal)	684	683

Total fat		
g	41.8	41.9
En%	55	55

Carbohydrates		
g	54.4	56.0
En%	32	33

Proteins		
g	20.3	20.3
En%	12	12

Values are based on package information. ^*∗*^ *Lycium barbarum* meal contained 25 g dried *Lycium barbarum*, providing 82 kcal, 0.9 g fat, 13.3 g carbohydrates, and 3.3 g protein.

**Table 2:** Baseline characteristics of the overweight men (*n*=17).

	Mean ± SD
Age (y)	59.5 ± 5.4
BMI (kg/m^2^)	27.2 ± 1.4
Weight (kg)	86.5 ± 6.5
Glucose (mmol/L)	5.3 ± 0.4
Total cholesterol (mmol/L)	5.3 ± 0.7
Triacylglycerol (mmol/L)	1.2 ± 0.4

## Data Availability

Data are included in the article and supplemental materials.

## Supplementary Materials

Supplementary Materials Supplemental Figure 1: flow chart of inclusion and exclusion of participants throughout the study. Supplemental Figure 2: mean changes in postprandial oxygen consumption and carbon dioxide production. Supplemental Figure 3: mean changes in postprandial fat and carbohydrate oxidation. Supplemental Table 1: habitual dietary intake of the men who participated in the trial as determined by a validated food frequency questionnaire. Supplemental Table 2: iAUC/dAUC over four hours for energy expenditure, respiratory quotient, and concentrations of plasma glucose, serum-free fatty acids, and serum triacylglycerol.
