# Supplementary material for: A Single Dose of Goji Berries Does Not Affect Postprandial Energy Expenditure and Substrate Oxidation in Healthy, Overweight Men
Source: J Nutr Metab. 2019 Feb 24;2019:4057143. doi: 10.1155/2019/4057143 (PMC6408998; doi:10.1155/2019/4057143)
Supplement: Supplementary Materials — Supplemental Figure 1: flow chart of inclusion and exclusion of participants throughout the study. Supplemental Figure 2: mean changes in postprandial oxygen consumption and carbon dioxide production. Supplemental Figure 3: mean changes in postprandial fat and carbohydrate oxidation. Supplemental Table 1: habitual dietary intake of the men who participated in the trial as determined by a validated food frequency questionnaire. Supplemental Table 2: iAUC/dAUC over four hours for energy expenditure, respiratory quotient, and concentrations of plasma glucose, serum-free fatty acids, and serum triacylglycerol. [file 4057143.f1.pdf]

A single dose of goji berries does not affect postprandial energy expenditure and substrate oxidation in healthy, overweight men

- Supplemental materials -

José J van den Driessche, Jogchum Plat, Guy Plasqui, Ronald P Mensink

Department of Nutrition and Movement Sciences, NUTRIM School for Nutrition and Translational Research in Metabolism, Maastricht University Medical Center+ (MUMC+), Maastricht, the Netherlands.

Corresponding author:

Ms. José J van den Driessche

Department of Nutrition and Movement Sciences

NUTRIM School for Nutrition and Translational Research in Metabolism

Maastricht University Medical Center+

P.O. Box 616

6200 MD Maastricht, the Netherlands

Email: j.vandendriessche@maastrichtuniversity.nl

Phone: +31 (0) 43 3881313

SUPPLEMENTAL TABLES AND FIGURES

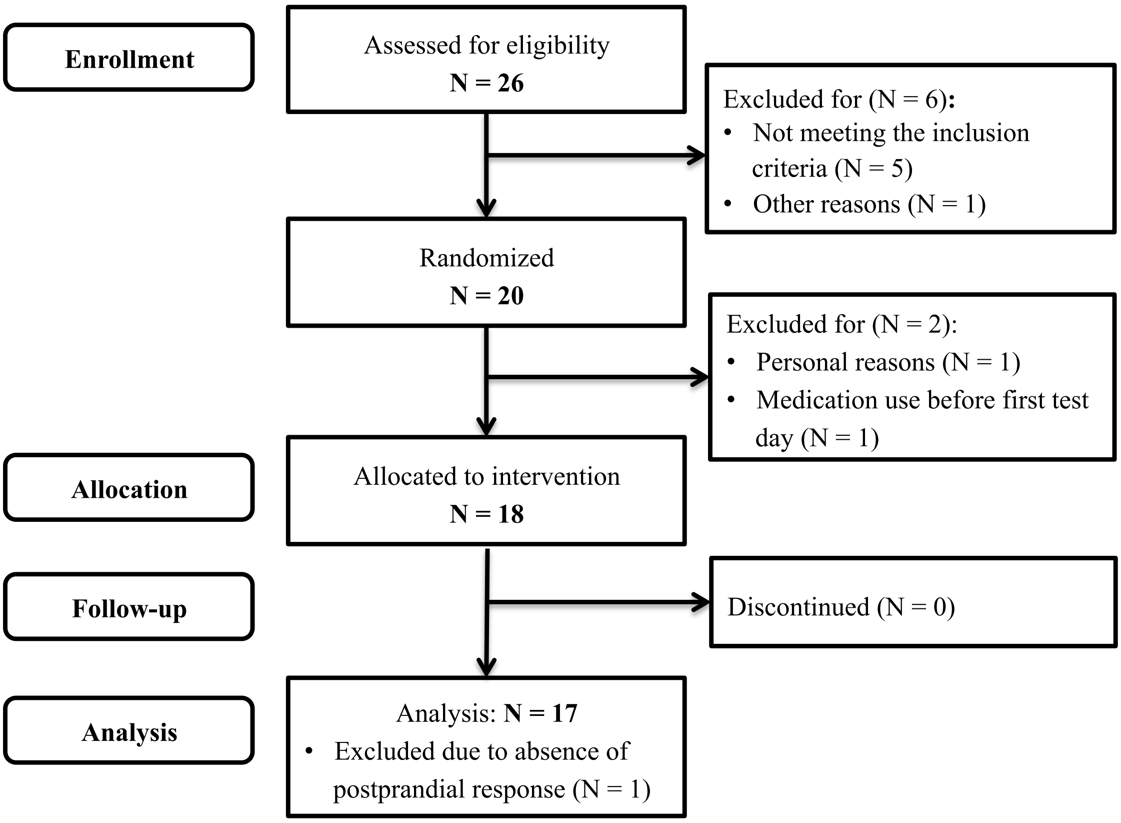

Supplemental figure 1: Flow chart of inclusion and exclusion of participants throughout this randomized, crossover study.

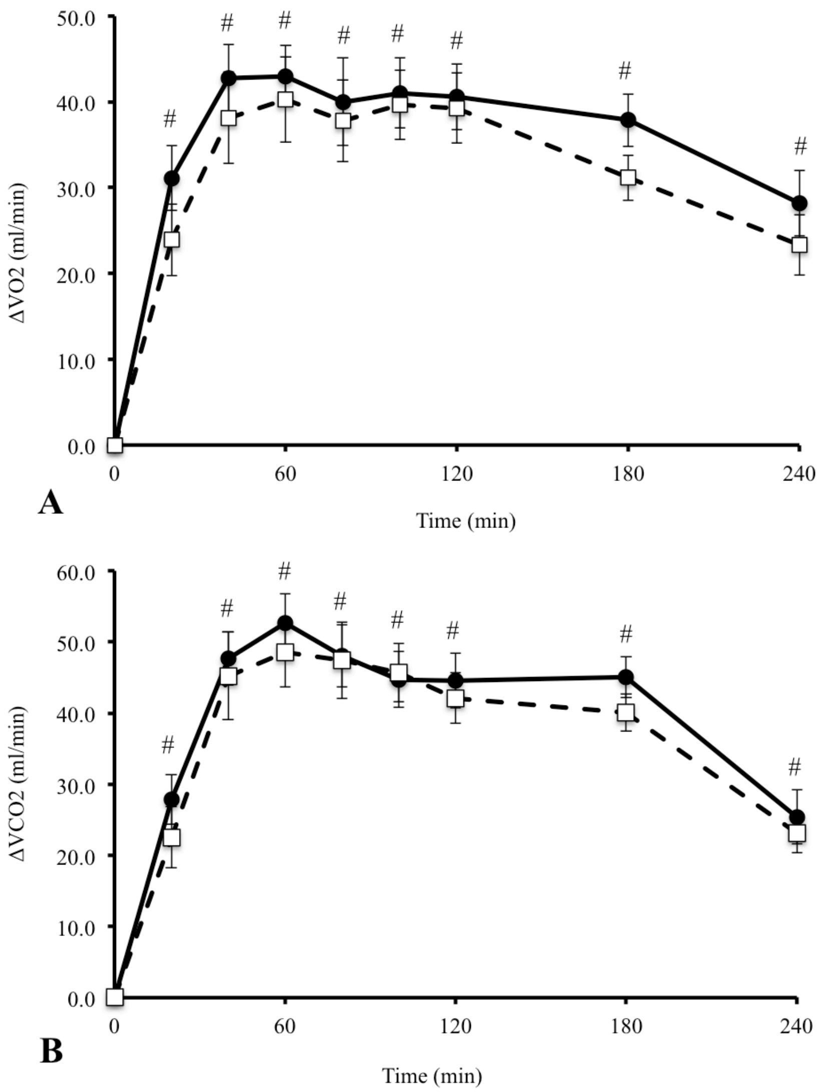

Supplemental figure 2: Mean changes (± SEM) in oxygen consumption ($VO_{2;}$ panel A) and carbon dioxide production (VCO_2; panel B) following the *Lycium barbarum* meal (□) and the control meal (•) in 17 healthy overweight men. Data was analyzed using linear mixed models. Significant effects ($P < 0.001$, with Bonferroni correction) were found for factor time (#) compared to baseline.

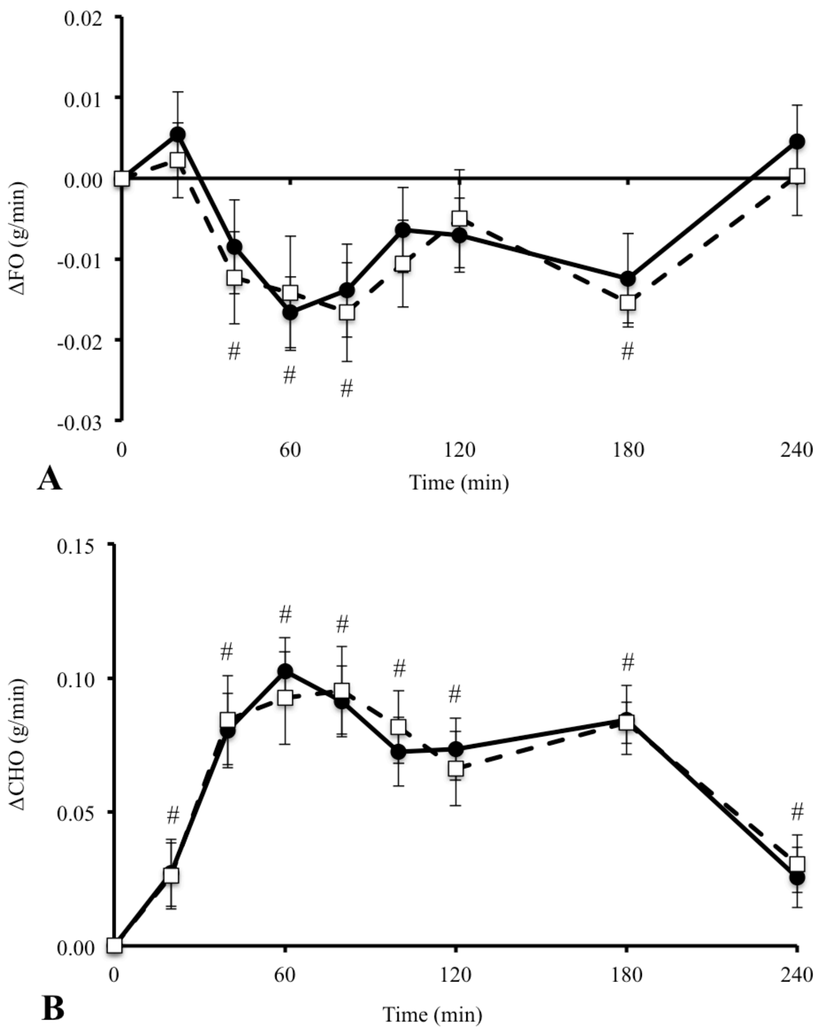

Supplemental figure 3: Mean changes (± SEM) in fat oxidation (FO; panel A) and carbohydrate oxidation (CHO; panel B) following the *Lycium barbarum* meal (□) and the control meal (•) in 17 healthy overweight men. Data was analyzed using linear mixed models. Significant effects (P < 0.001, with Bonferroni correction) were found for factor time (#) compared to baseline.

Supplemental table 1: habitual dietary intake of the men who participated in the trial as determined by a validated food frequency questionnaire.[1]

Energy (kcal/day)	2544 (1773 – 4425)
Fat (energy %)	35.6 (26.7 – 41.1)
SFA	12.5 (8.7 – 14.0)
MUFA	12.2 (8.2 – 16.9)
PUFA	7.3 (4.9 – 10.4)
Protein (energy %)	16.2 (11.9 – 19.7)
Carbohydrates (energy %)	43.8 (28.1 – 50.1)
Alcohol (energy %)	3.2 (0.0 – 12.7)
Fiber (g/day)	31.4 (20.1 – 72.3)
Cholesterol (mg/day)	270 (123 – 435)

[1] Data is presented as medians with ranges

Supplemental table 2: iAUC/dAUC over 4 hours for, energy expenditure (EE), respiratory quotient (RQ), and concentrations of plasma glucose, serum free fatty acids (FFA) and serum triacylglycerol (TAG) after *Lycium barbarum* and control meal intake[1]

			***Lycium barbarum* meal**	**Control meal**
EE	kJ per 240 min	iAUC	167 (90 – 269)	176 (126 – 298)
RQ	per 240 min	iAUC	10.6 (0.0 – 18.9)	8.9 (1.5 – 21.8)
Glucose	mmol /L per 240 min	iAUC	58.4 (6.1 – 225.4)	64.0 (3.5 – 290.3)
FFA	mol*10^3 /L per 240 min	dAUC	26.9 (0.9 – 57.8)	16.8 (3.2 – 43.9)
TAG	mmol /L per 240 min	iAUC	108 (25 – 202)	101 (29 – 251)

[1] Data is presented as medians with ranges.
